# Supplementary material for: Molecular and Polymer Ln2M2 (Ln = Eu, Gd, Tb, Dy; M = Zn, Cd) Complexes with Pentafluorobenzoate Anions: The Role of Temperature and Stacking Effects in the Structure; Magnetic and Luminescent Properties
Source: Materials (Basel). 2020 Dec 13;13(24):5689. doi: 10.3390/ma13245689 (PMC7763275; doi:10.3390/ma13245689)
Supplement: Supplementary file 1 [file materials-13-05689-s001.pdf]

materials

Supplementary Materials

Molecular and Polymer Ln_2M_2 (Ln = Eu, Gd, Tb, Dy; M = Zn, Cd) Complexes with Pentafluorobenzoate Anions: The Role of Temperature and Stacking Effects in the Structure; Magnetic and Luminescent Properties

Maxim A. Shmelev [1], Mikhail A. Kiskin [1,*], Julia K. Voronina [1], Konstantin A. Babeshkin [1], Nikolay N. Efimov [1], Evgenia A. Varaksina [2], Vladislav M. Korshunov [2,3], Ilya V. Taydakov [2,4], Natalia V. Gogoleva [1], Alexey A. Sidorov [1] and Igor L. Eremenko [1]

1 N. S. Kurnakov Institute of General and Inorganic Chemistry, Russian Academy of Sciences, 119991 Moscow, Russia; shmelevma@yandex.ru (M.A.S.); juliavoronina@mail.ru (J.K.V.); bkonstantan@yandex.ru (K.A.B.); nnefimov@yandex.ru (N.N.E.); judiz@rambler.ru (N.V.G.); sidorov@igic.ras.ru (A.A.S.); ilerem@igic.ras.ru (I.L.E.)
2 P. N. Lebedev Physical Institute, Russian Academy of Sciences, 119991 Moscow, Russia; janiy92@yandex.ru (E.A.V.); vladkorshunov@bk.ru (V.M.K.); taidakov@mail.ru (I.V.T.)
3 Faculty of Fundamental Sciences, Bauman Moscow State Technical University, 105005 Moscow, Russia
4 Academic Department of Innovational Materials and Technologies Chemistry, Plekhanov Russian University of Economics, 117997 Moscow, Russia
* Correspondence: mkiskin@igic.ras.ru

Received: 12 November 2020; Accepted: 8 December 2020; Published: date

I. Structural data
II. PXRD data
III. Photoluminescence data
IV. Magnetic data

I. Structural data

Table S1. SHAPE analysis for compound I–X.

-	M	Ln
I	Capped trigonal prism (2.080)<br>Capped octahedron (2.787)	Muffin (1.718)<br>Spherical capped square antiprism (2.032)<br>Capped square antiprism J10 (2.335)
II	Capped trigonal prism (2.051)<br>Capped octahedron (2.837)	Muffin (1.619)<br>Spherical capped square antiprism (1.920)<br>Capped square antiprism J10(2.218)
III	Capped trigonal prism (2.015)<br>Capped octahedron (2.829)	Muffin (1.621)<br>Spherical capped square antiprism (1.900)<br>Capped square antiprism J10 (2.259)
IV	Capped trigonal prism (2.020)<br>Capped octahedron (2.841)	Muffin (1.619)<br>Spherical capped square antiprism (1.921)<br>Capped square antiprism J10(2.273)
V	Pentagonal bipyramid (5.509)<br>Capped trigonal prism (6.460)	Triangular dodecahedron (1.291)<br>Biaugmented trigonal prism (2.226)<br>Square antiprism (2.396)
VI	Octahedron (3.190)	Biaugmented trigonal prism (1.576)

		Triangular dodecahedron (1.991)<br>Biaugmented trigonal prism J50 (1.995)
VII	Octahedron (2.877)	Capped square antiprism J10 (1.051)<br>Muffin (1.239)<br>Capped square antiprism J10 (1.736)
VIII	Octahedron (3.110)	Biaugmented trigonal prism (1.620)<br>Triangular dodecahedron (1.911)<br>Biaugmented trigonal prism J50 (1.980)
IX	Octahedron (3.110)	Triangular dodecahedron (1.605)<br>Biaugmented trigonal prism (1.766)<br>Biaugmented trigonal prism J50 (2.273)
X	Trigonal prism (3.792)	Square antiprism (0.480)<br>Biaugmented trigonal prism (1.753)<br>Triangular dodecahedron (2.214)

Table S2. Stacking interactions in the crystal packing of I–X.

Interaction	Symmetry code	Cg-Cg, Å	Cg-Perp, Å	A/deg
Complex I				
phen-pfb	-	3.562(4)	3.363(3)	4.8(4)
phen-pfb	-	3.618(5)	3.481(3)	5.6(4)
pfb-pfb	-	3.724(5)	3.290(4)	4.7(4)
Complex II				
phen-pfb	-	3.565(5)	3.368(4)	4.6(4)
phen-pfb	-	3.632(5)	3.487(4)	5.6(4)
pfb-pfb	-	3.743(5)	3.311(4)	4.5(4)
Complex III				
phen-pfb	-	3.625(5)	3.486(4)	6.1(4)
phen-pfb	-	3.552(5)	3.349(4)	4.8(4)
pfb-pfb	-	3.720(5)	3.298(4)	4.9(4)
Complex IV				
phen-pfb	-	3.551(9)	3.444(7)	5.9(7)
phen-pfb	-	3.602(8)	3.352(7)	4.3(7)
phen-pfb	-	3.455(8)	3.368(4)	4.5(7)
phen-pfb	-	3.774(9)	3.385(4)	4.7(8)
pfb-pfb	-	3.773(6)	3.349(5)	5.2(5)
Complex V				
phen-phen	1-X,-Y,-Z	3.430(3)	3.387(2)	0.0(3)
pfb-pfb	1-X,-Y,1-Z	3.716(4)	3.496(3)	0.0(3)
Complex VI				
phen-phen	2-X,1-Y,-Z	3.411(3)	3.399(3)	0.4(2)
pfb-pfb	2-X,1-Y,-Z	3.707(4)	3.400(3)	0.9(3)
Complex VII				
phen-phen	2-X,1-Y,-Z	3.663(2)	3.6142(1)	0.76(1)
pfb-pfb	1-X,1-Y,1-Z	3.628(2)	3.4326(1)	0.02(1)
Complex VIII				
phen-phen	-X,1-Y,2-Z	3.434(4)	3.424(2)	0.6(2)
pfb-pfb	-X,1-Y,2-Z	3.723(4)	3.426(2)	1.3(2)
Complex IX				
phen-phen	2-X,1-Y,1-Z	3.598(3)	3.446(1)	0.02(17)
pfb-pfb	2-X,1-Y,1-Z	3.719(3)	3.449(1)	0.81(17)
Complex X				
phen-phen	-	3.531(9)	3.522(6)	5.5(7)
phen-phen	1/2+X,1/2-Y,1/2+Z	3.514(7)	3.506(5)	4.2(4)
phen-pfb	-	3.551(12)	3.495(7)	8
pfb-pfb	1/2-X,3/2-Y,1-Z	3.599(11)	3.404(8)	0.0(9)

Note. Cg is the centroid of aromatic rings, Perp is the perpendicular to the ring plane, α is the angle between the planes of aromatic moieties.

Table S3. C-H…X (X= O, N, F) interactions in the crystal packing of I–X.

Interaction	D-H, Å	H...A, Å	D...A, Å	D-H...A, °
Complex I				
C36-H36...F10A	0.95	2.53	3.376(10)	149
C43-H43...F13A	0.95	2.45	3.081(12)	124
C43-H43...F13B	0.95	2.41	3.028(15)	123
C45-H45...F28	0.95	2.51	3.128(8)	123
C51-H51B...F11A	0.98	2.50	3.37(2)	148
C53-H53C...F22	0.98	2.49	3.445(13)	165
C41-H41...N4	0.95	2.55	3.461(14)	161
C53-H53A...O7	0.98	2.59	3.437(12)	145
C53-H53C...O5	0.98	2.57	3.131(13)	116
Complex II				
C36-H36...F10A	0.93	2.20	3.092(16)	161
C42-H42...F13B	0.93	2.34	2.959(17)	124
C44-H44...F31	0.93	2.52	3.125(9)	123
C51-H51A...F21	0.96	2.53	3.455(14)	163
C48-H48...N3	0.93	2.56	3.450(16)	161
C51-H51A...O5	0.96	2.44	3.142(14)	130
C51-H51C...O10	0.96	2.58	3.418(14)	146
Complex III				
C36-H36...F24	0.95	2.51	3.120(8)	122
C38-H38...F13A	0.95	2.31	2.932(17)	122
C45-H45...F10B	0.95	2.17	3.074(15)	159
C40-H40...N4A	0.95	2.38	3.288(19)	159
C51-H51A...N4B	0.98	2.38	3.20(3)	140
C51-H51C...N4B	0.98	2.54	2.45(3)	154
C53B-H53D..O6	0.98	2.46	3.149(16)	127
C53B-H53F..O8	0.98	2.58	3.332(15)	132
Complex IV				
C36A-H36A...F10A	0.95	1.97	2.85(2)	153
C37A-H37A...F32	0.95	2.37	3.313(18)	169
C43A-H43A...F13B	0.95	2.41	3.00(3)	120
C36A-H36A...O4	0.95	2.51	3.098(18)	120
C51-H51B...N4	0.98	2.58	3.36(3)	137
C53-H53C...O5	0.98	2.31	3.125(15)	140
Complex V				
C47-H47...F4	0.93	2.46	3.375(7)	167
C45-H45...O4	0.93	2.42	3.191(8)	140
Complex VI				
C51-H51B...F18	0.96	2.45	3.215(12)	137
C43-H43...O4	0.93	2.51	3.268(8))	138
C53-H53C...O9	0.96	2.56	3.348(11)	140
Complex VII				
C53-H53A...F17	0.96	2.40	3.162(7)	136
O1W-H1WA…O9	0.86	2.35	2.630(3)	100
O1W-H1WB…N4	0.85	2.38	2.829(5)	113
C45-H45...O2	0.93	2.59	3.110(3)	116
C53-H53A...N3	0.96	2.53	3.424(7)	154
Complex VIII				
C53-H53B...F20	0.98	2.43	3.207(9)	136
C38-H38...O2	0.95	2.53	3.283(6)	137
C51-H51C...O7	0.98	2.58	3.359(8)	137

Complex IX				
C45-H45...F32B	0.95	2.50	3.131(9)	124
C37-H37...F12A	0.95	2.54	3.421(8)	154
C51A-H51B...F18	0.98	2.35	3.181(11)	142
C43-H43...O4	0.95	2.60	3.369(4)	139
C51A-H51C...N3B	0.98	2.56	2.469(16)	151
C53A-H53E...O9	0.98	2.53	3.449(16)	156
C53A-H53F...N4A	0.98	1.89	2.697(19)	138
Complex X				
C43-H43...F13	0.93	2.40	3.259(17)	154
C60-H60A...F12	0.96	2.55	3.31(3)	136
C45-H45...N6	0.93	2.57	3.41(3)	150
C52-H52...O9	0.93	2.59	3.110(17)	116

Table S4. C-X… π (X= N, F) interactions in the crystal packing of I–X.

Interaction	F…Cg, Å	F-Perp, Å	Gamma, °	C-F…Cg, °	C…Cg, Å
Complex I					
C5-F5…Cg	3.555(5)	−3.058	24.31	154.2(4)	4.584(7)
C7-F7…Cg	3.262(6)	3.229	8.22	100.6(3)	3.749(7)
C12A-F12A…Cg	3.288(11)	3.198	13.42	118.2(5)	4.092(9)
C12B-F12B…Cg	3.268(15)	3.138	25.93	118.9(10)	4.102(14)
C28-F28…Cg	3.356(4)	3.188	18.21	139.4(3)	4.455(6)
C31-F31…Cg	3.260(6)	−3.230	7.76	99.0(4)	3.715(8)
C32-F32…Cg	3.554(6)	−3.196	25.93	67.9(4)	3.294(8)
C33-F33…Cg	3.471(7)	−3.287	18.72	73.1(5)	3.338(10)
C52-N4…Cg	3.443(13)	−3.187	22.22	101.2(9)	3.814(12)
Complex II					
C5-F5…Cg	3.338(6)	−3.049	24.00	155.0(5)	4.589(7)
C7-F7…Cg	3.255(6)	−3.225	7.84	100.4(4)	3.739(8)
C12A-F12A…Cg	3.62(2)	−3.440	18.23	120.8(15)	4.44(2)
C12B-F12B…Cg	3.194(14)	−3.121	12.21	117.2(8)	3.998(11)
C24-F24…Cg	3.258(6)	3.235	7.37	98.6(4)	3.703(8)
C25-F25…Cg	3.568(7)	3.196	26.42	67.0(4)	3.284(9)
C26-F26…Cg	3.489(8)	3.317	18.10	73.2(5)	3.355(10)
C31-F31…Cg	3.356(5)	−3.187	18.29	139.8(3)	4.466(7)
Complex III					
C3-F3…Cg	3.253(6)	−3.220	8.24	100.5 (9)	3.740(7)
C5-F5…Cg	3.350(5)	−3.063	23.88	153.9(4)	4.589(6)
C12A-F12A…Cg	3.350(5)	−3.073	12.82	117.9(9)	3.966(10)
C24-F24…Cg	3.332(4)	−3.160	18.50	139.1(3)	4.440(7)
C31-F31…Cg	3.332(4)	−3.160	18.50	98.4(5)	3.386(10)
C32-F32…Cg	3.549(6)	3.181	26.31	67.0(4)	3.267(10)
C33-F33…Cg	3.497(7)	3.316	18.53	72.1(5)	3.338(10)
C52B-N4B…Cg	3.35(2)	3.310	8.88	84	3.40(3)
C52A-N4A…Cg	3.11(2)	2.940	18.72	106.8(14)	3.72(3)
Complex IV					
C5-F5…Cg	3.319(6)	−3.048	23.30	157.3(6)	4.584(9)
C6-F6…Cg	3.622(8)	3.200	27.93	118.3(5)	4.429(10)
C7-F7…Cg	3.238(12)	3.166	12.18	106.3(5)	3.841(14)
C12B-F12B…Cg	3.15(2)	3.020	16.49	120.4(12)	4.014(15)
C33-F33…Cg	3.535(9)	−3.386	16.74	73.0(6)	3.395(13)
C34-F34…Cg	3.517(9)	−3.169	25.66	68.2(5)	3.264(13)
C35-F35…Cg	3.261(9)	−3.225	8.54	90.6(5)	3.540(11)
Complex V					
C4-F4…Cg	3.249(5)	3.223	7.13	95.1(4)	3.626(8)

C18A-F36A…Cg	3.39(2)	3.200	18.91	151.1(18)	4.610(10)
C20A-F38A…Cg	3.45(3)	3.330	15.25	138(3)	4.527(14)
C18B-F18B…Cg	3.26(3)	−3.220	9.08	111.9(18)	3.950(17)
C19B-F19B…Cg	3.19(2)	3.020	18.76	139.9(16)	4.252(15)
C20B-F20B…Cg	3.41(4)	−3.210	19.39	119(2)	4.21(2)
C24-F24…Cg	3.557(5)	−3.473	12.49	88.8(3)	3.774(7)
C27-F27…Cg	3.440(7)	3.070	26.83	133.3(5)	4.474(8)
C35-F35…Cg	3.659(10)	−3.300	25.63	97.0(4)	4.047(11)
Complex VI					
C19-F19…Cg	3.289(6)	−3.017	23.45	95.6(4)	3.671(9)
C19-F19…Cg	2.988(5)	2.920	10.71	157.5(4)	4.240(7)
C20-F20…Cg	3.195(5)	3.114	13.02	123.6(5)	4.096(7)
C26-F26…Cg	3.459(5)	−3.223	21.29	135.3(5)	4.502(8)
C35-F35…Cg	3.054(4)	2.992	11.54	135.1(4)	4.106(7)
Complex VII					
C4-F4…Cg	3.205(2)	−3.196	4.20	89.26(17)	3.459(3)
C18-F18…Cg	3.127(3)	−3.090	8.80	107.98(16)	3.761(3)
C19-F19…Cg	3.078(3)	−3.027	10.46	140.4(3)	4.197(5)
C20-F20…Cg	3.383(3)	−3.121	22.69	118.4(2)	4.190(5)
C25-F25…Cg	3.421(3)	3.129	23.85	96.4(2)	3.810(4)
C26-F26…Cg	3.266(3)	3.244	6.64	89.1(2)	3.510(5)
C35-F35…Cg	3.548(2)	3.530	5.76	87.24(16)	3.733(4)
Complex VIII					
C18-F18…Cg	3.214(5)	−3.128	13.27	124.0(4)	4.127(6)
C19-F19…Cg	2.971(4)	−2.934	8.99	134.1(3)	4.028(6)
C20-F20…Cg	3.303(5)	3.029	23.51	94.8(3)	3.665(6)
C25-F25…Cg	3.398(5)	−2.973	28.98	137.0(3)	4.476(7)
C28-F28…Cg	3.060(4)	−2.994	11.91	135.8(3)	4.132(6)
C33-F33…Cg	3.497(5)	3.263	21.11	135.2(4)	4.556(7)
Complex IX					
C13A-F13A…Cg	3.755(11)	−3.313	28.05	134.2(6)	4.780(10)
C19-F19…Cg	3.182(4)	2.994	19.82	136.9(4)	4.261(6)
C20-F20…Cg	3.212(5)	3.094	15.60	124.8(3)	4.132(5)
C21-F21…Cg	3.457(4)	−3.088	26.69	119.5(2)	4.277(6)
C28-F28…Cg	3.655(4)	3.230	27.91	130.5(2)	4.636(5)
C32A-F32A…Cg	3.652(12)	3.283	25.99	136.8(4)	4.708(12)
C35A-F35A…Cg	3.215(6)	−3.019	20.13	141.9(4)	4.333(6)
C35B-F35B…Cg	3.872(7)	3.719	16.14	98.4(4)	4.262(7)
Complex X					
C13-F13…Cg	3.309(12)	3.015	24.35	94.8(10)	3.66(2)
C23-F23…Cg	3.342(14)	3.335	3.66	93.4(10)	3.655(18)
C56-F32…Cg	3.457(12)	−3.152	24.23	130.6(10)	4.449(17)

Table S5. Crystallographic parameters and structure refinement statistics for I–V.

Compound	I	II	III	IV	V
Formula	$C_{50}H_{12,5}CdEuF_{25}N_{3,5}O_{10}$	$C_{50}H_{12,5}CdGdF_{25}N_{3,5}O_{10}$	$C_{50}H_{12,5}CdTbF_{25}N_{3,5}O_{10}$	$C_{50}H_{12,5}CdDyF_{25}N_{3,5}O_{10}$	$C_{94}H_{16}Cd_2Tb_2F_{50}N_4O_{20}$
M	1561.49	1566.78	1568.45	1572.03	2436.39(18)
T (K)	150(2)	296(2)	150(2)	150(2)	296(2)
Crystal system	Triclinic	Triclinic	Triclinic	Triclinic	Triclinic
Space group	$P\bar{1}$	$P\bar{1}$	$P\bar{1}$	$P\bar{1}$	$P\bar{1}$
a (Å)	13.2782(6)	13.279(8)	13.234(5)	13.2177(16)	14.3290(6)
b (Å)	14.8919(11)	14.979(9)	14.899(5)	14.8889(19)	14.6711(6)
c (Å)	15.3726(8)	15.310(11)	15.323(6)	15.366(3)	14.9048(6)
α (°)	92.834(2)	92.42(4)	92.586(13)	92.362(5)	98.5850(10)
β (°)	104.393(2)	104.24(3)	104.484(14)	104.101(5)	114.4100(10)
γ (°)	116.023(2)	116.29(2)	115.948(10)	116.169(5)	113.098(2)
V (Å^3)	2601.6(3)	2606(3)	2588.7(16)	2593.6(6)	2436.39(18)
Z	2	2	2	2	1
$D_{calc.}$ (g cm^{-3})	1.993	1.997	2.012	2.013	2.054
μ (mm^{-1})	1.756	1.822	1.919	1.993	2.034
θ_{max} (°)	26.04	26.10	26.00	26.00	26.00
T_{min}/T_{max}	0.720/0.844	0.5686/ 0.7453	0.762/0.877	0.6002/0.7453	0.6044/0.7465
Reflections collected	16819	21347	20361	16708	24135
Independent reflections collected	9839	10138	10049	9882	9526
Reflections with $I > 2\sigma(I)$	8464	9205	8771	7675	8217
R_{int}	0.0311	0.0212	0.0251	0.0691	0.0524
GOOF	1.052	1.042	1.043	1.035	1.049
R_1 ($I > 2\sigma(I)$)	0.0440	0.0457	0.0449	0.0532	0.0385
wR_2 ($I > 2\sigma(I)$)	0.0525	0.0501	0.0525	0.0720	0.0473
$\Delta\rho_{min}/\Delta\rho_{max}$ (e/Å^3)	−2.056/1.864	−1.509/2.873	−1.435/2.323	−1.869/2.312	−1.229/0.487

Table S6. Crystallographic parameters and structure refinement statistics for VI–X.

Compound	VI	VII	VIII	IX	X
Formula	$C_{102}H_{28}Zn_2Eu_2F_{50}N_8O_{20}$	$C_{102}H_{32}Zn_2Gd_2F_{50}N_8O_{22}$	$C_{102}H_{28}Zn_2Tb_2F_{50}N_8O_{20}$	$C_{102}H_{28}Zn_2Dy_2F_{50}N_8O_{20}$	$C_{126}H_{44}Cd_2Eu_2F_{50}N_{12}O_{20}$
M	3069.98	3116.59	3083.90	3091.06	3524.45
T (K)	150(2)	296(2)	150(2)	150(2)	296(2)
Crystal system	Triclinic	Triclinic	Triclinic	Triclinic	Monoclinic
Space group	$P\bar{1}$	$P\bar{1}$	$P\bar{1}$	$P\bar{1}$	C2/*c*
a (Å)	13.7674(9)	13.7618(6)	13.811(6)	14.062(6)	35.904(4)
b (Å)	14.2446(9)	14.0345(9)	14.259(11)	14.421(6)	14.6659(17)
c (Å)	15.7103(8)	16.0942(8)	15.791(8)	15.883(7)	30.104(3)
α (°)	66.556(2)	83.922(2)	66.31(3)	64.689(12)	90
β (°)	80.817(2)	66.3600(10)	80.566(16)	79.298(12)	119.866(4)
γ (°)	66.901(2)	67.6770(10)	66.46(3)	64.976(12)	90
V (Å^3)	2600.0(3)	2630.2(2)	2611(3)	2638.2(19)	13746(3)
Z	1	1	1	1	4
$D_{calc.}$ (g cm^{-3})	1.961	1.968	1.962	1.946	1.703
μ (mm^{-1})	1.811	1.861	1.957	2.012	1.341
θ_{max} (°)	26.00	26.15	26.00	26.01	24.40
T_{min}/T_{max}	0.773/0.840	0.707/0.836	0.508/0.696	0.689/0.824	0.689/0.775

Reflections collected	16376	18004	18431	19394	56608
Independent reflections collected	10130	10164	10026	10236	11305
Reflections with $I > 2\sigma(I)$	8015	9168	9167	9368	7162
R_{int}	0.1077	0.0230	0.0375	0.0216	0.1277
GOOF	0.992	1.024	1.065	1.036	0.905
R_1 ($I > 2\sigma(I)$)	0.0565	0.0298	0.0368	0.0304	0.0800
wR_2 ($I > 2\sigma(I)$)	0.0727	0.0351	0.0420	0.0346	0.1265
$\Delta\rho_{min}/\Delta\rho_{max}$ (e/Å^3)	−2.335/2.756	−0.696/0.995	−1.039/1.361	−0.688/1.053	−1.708/1.725

II. PXRD data

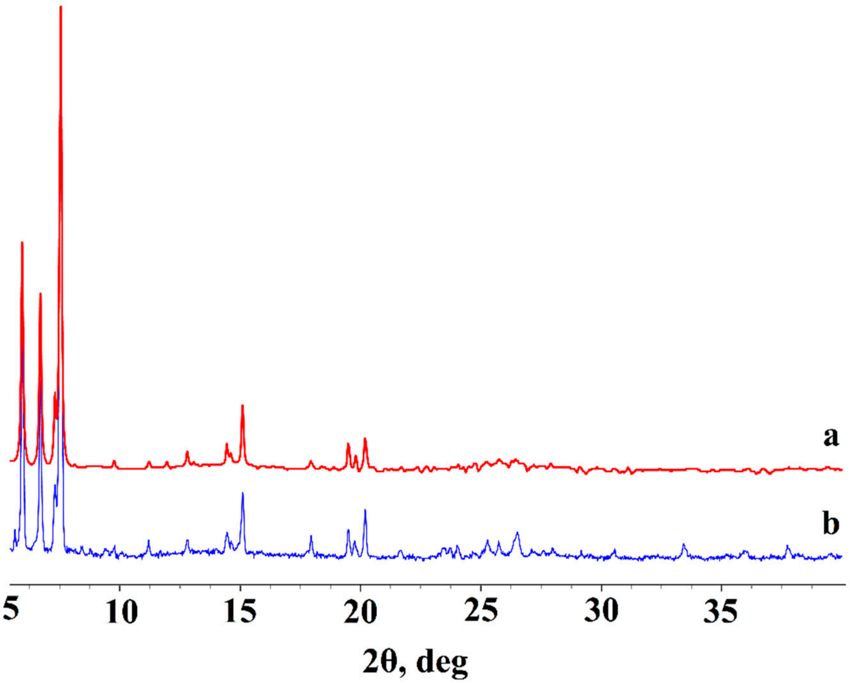

Figure S1. Calculated (a) and experimental (b) diffractograms for compound I.

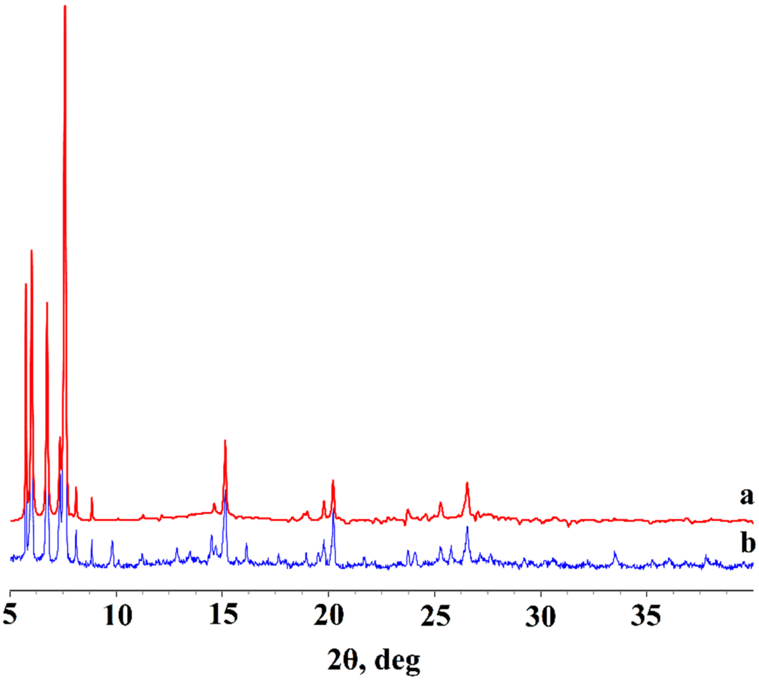

Figure S2. Calculated (a) and experimental (b) diffractograms for compound II.

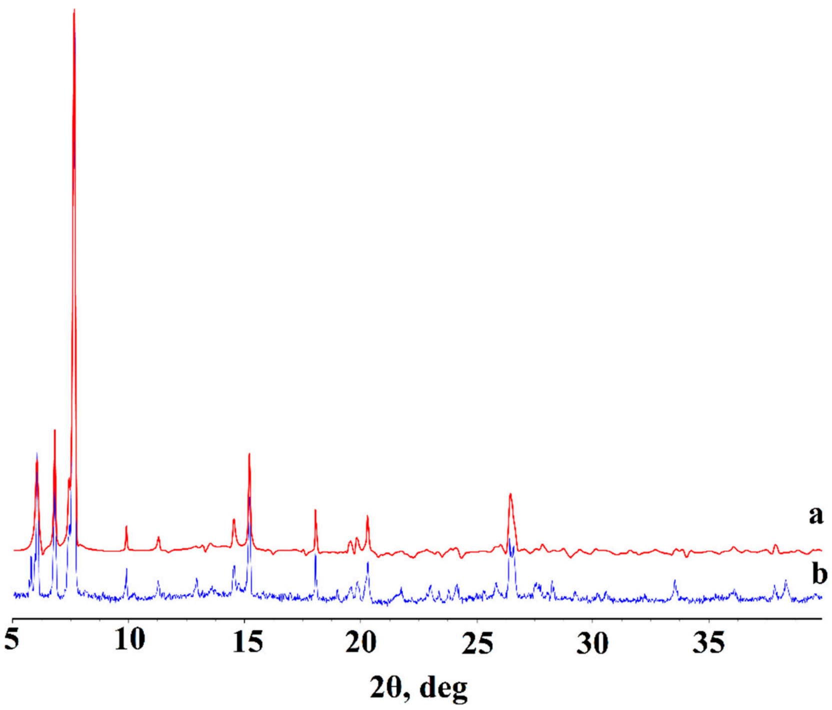

Figure S3. Calculated (a) and experimental (b) diffractograms for compound III.

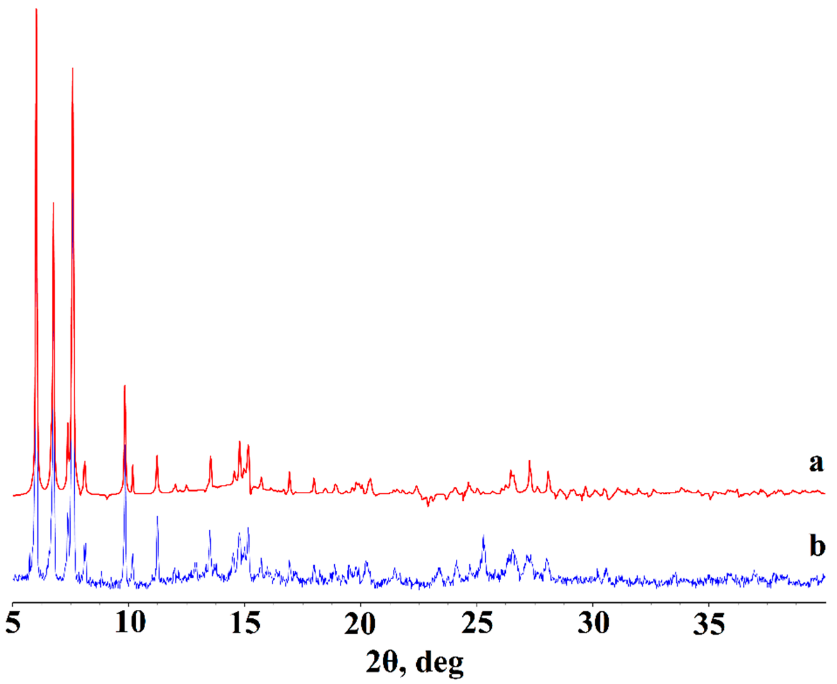

Figure S4. Calculated (a) and experimental (b) diffractograms for compound IV.

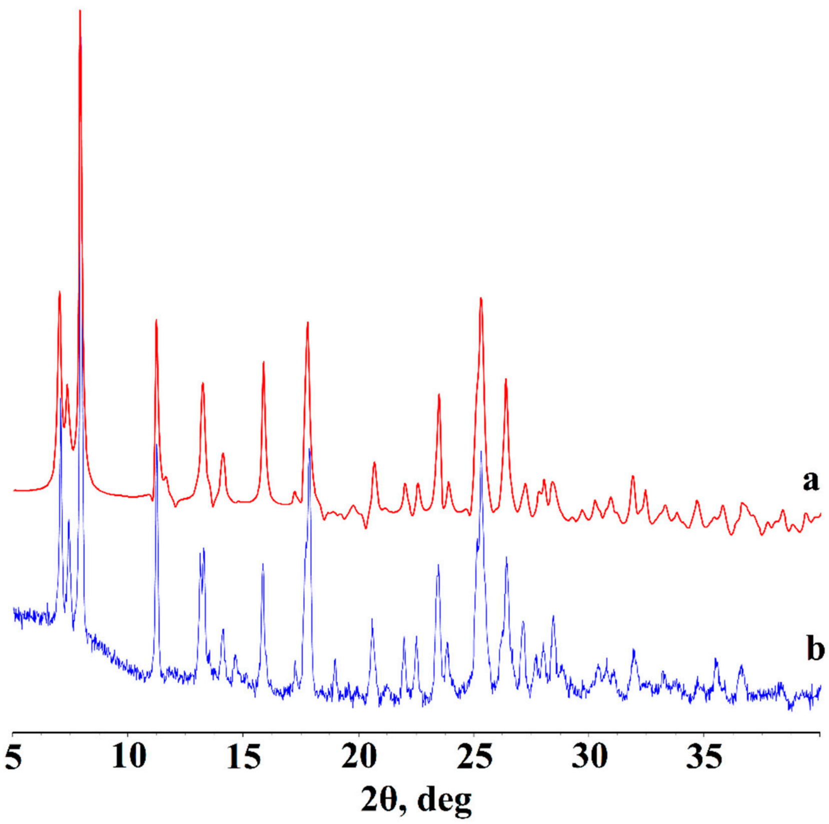

Figure S5. Calculated (a) and experimental (b) diffractograms for compound V.

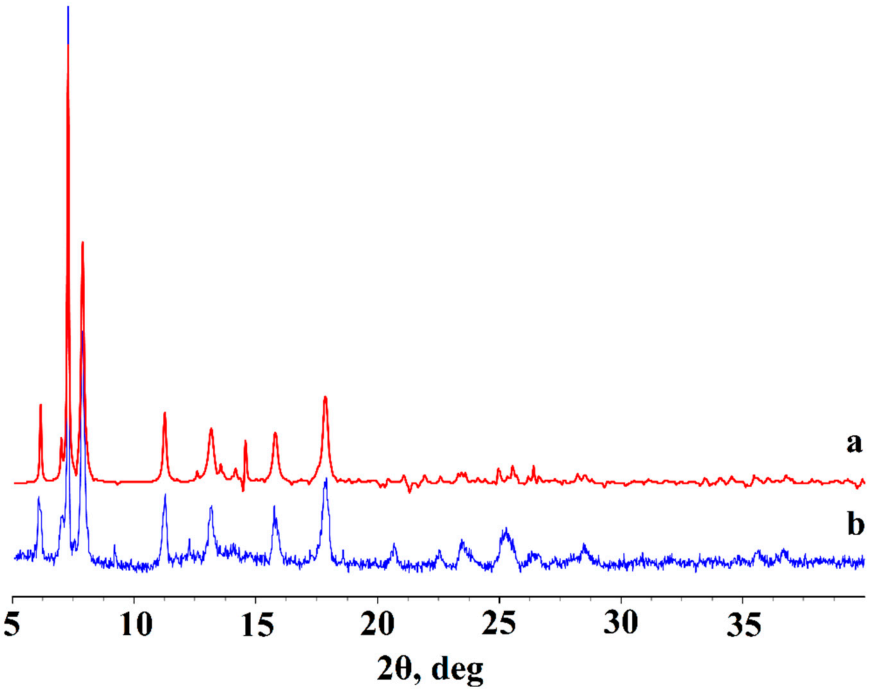

Figure S6. Calculated (a) and experimental (b) diffractograms for compound VI.

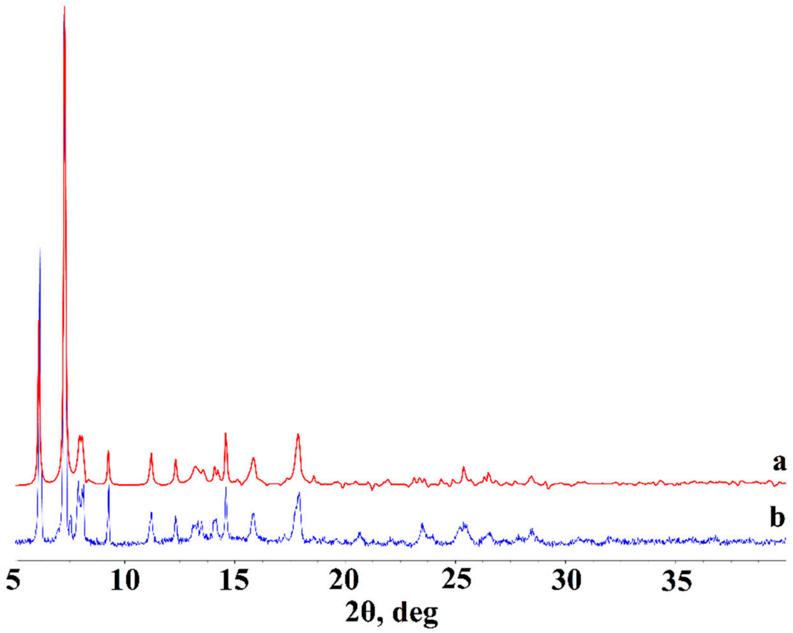

Figure S7. Calculated (a) and experimental (b) diffractograms for compound VII.

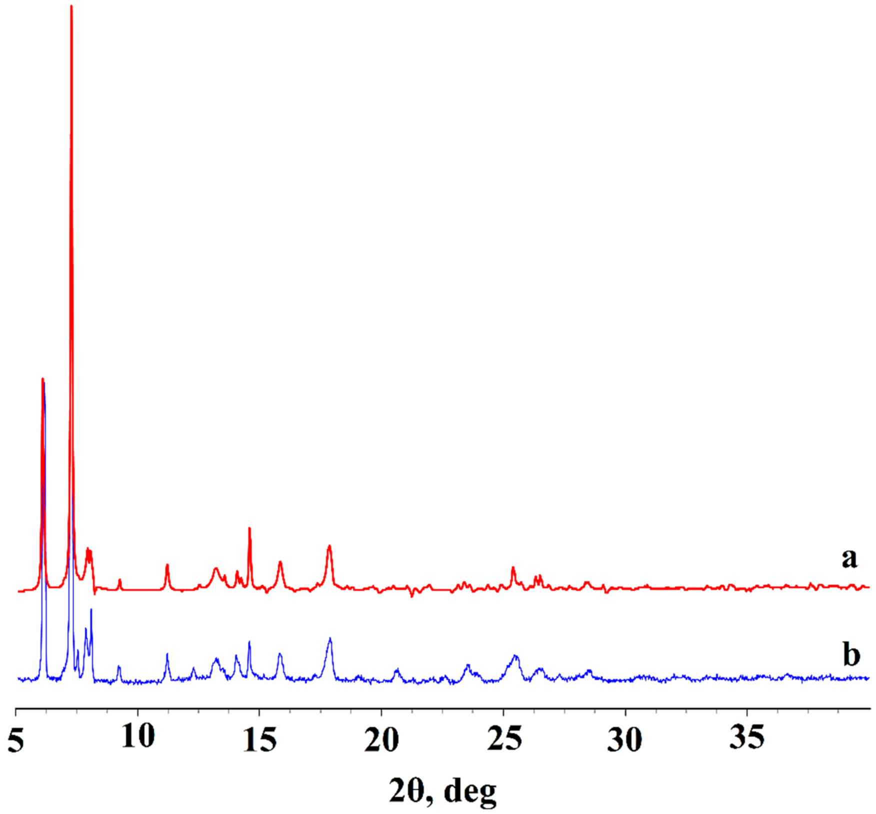

Figure S8. Calculated (a) and experimental (b) diffractograms for compound VIII.

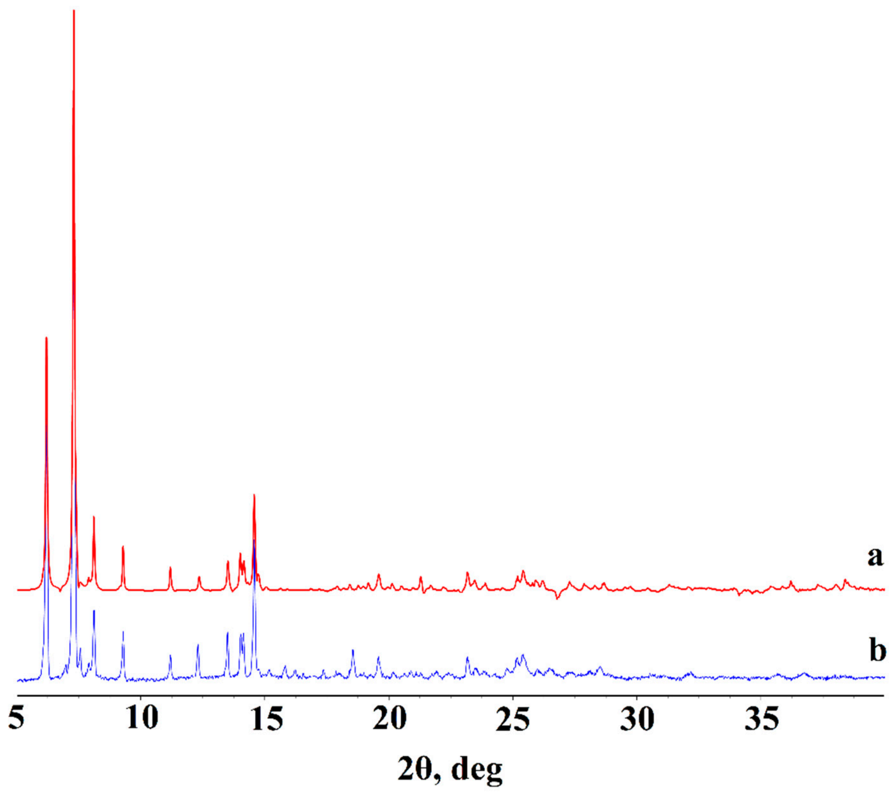

Figure S9. Calculated (a) and experimental (b) diffractograms for compound IX.

III. Photoluminescence data

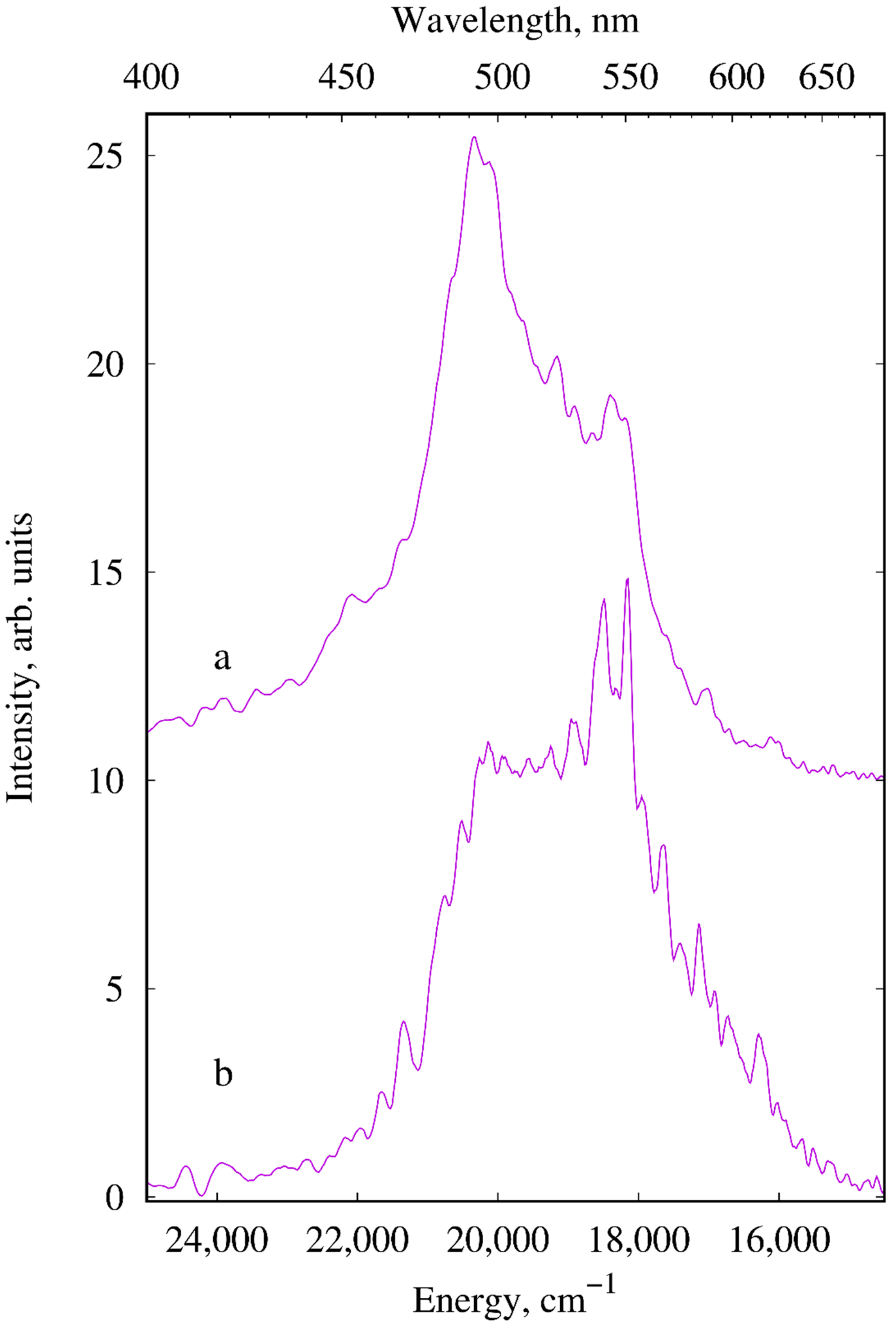

Figure S10. Phosphorescence spectra of II (a) and VII (b) (solid samples, λ_{ex} = 280 nm, *T* = 77 K).

IV. Magnetic data

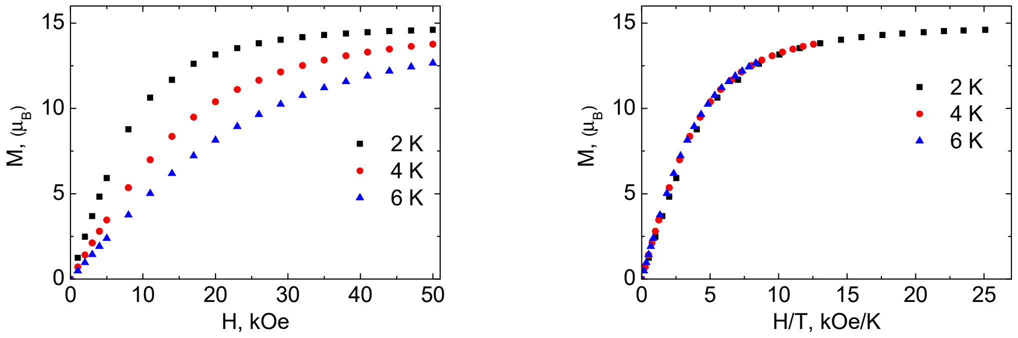

Figure S11. The M(H) and M(H/T) dependences at different temperatures for complex II.

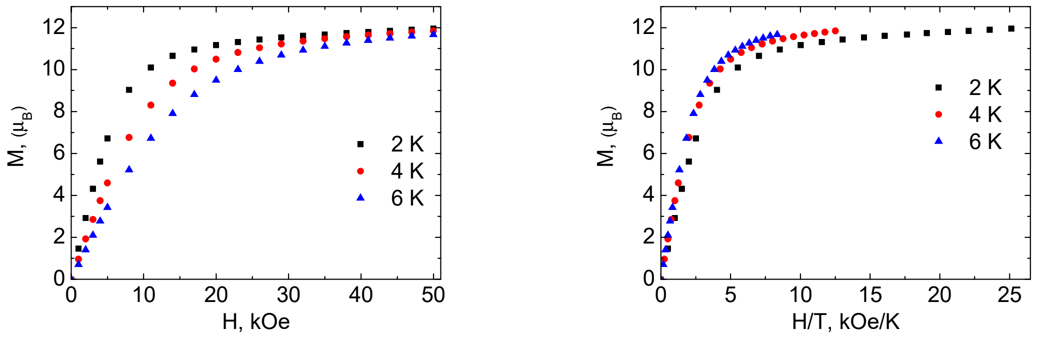

Figure S12. The M(H) and M(H/T) dependences at different temperatures for complex III.

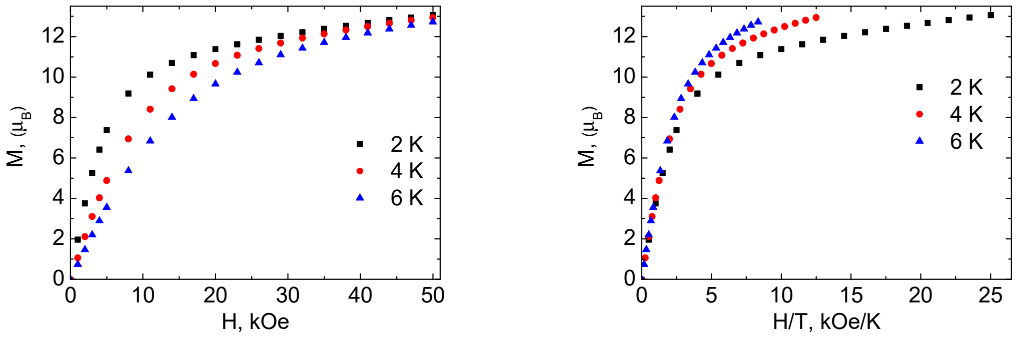

Figure S13. The M(H) and M(H/T) dependences at different temperatures for complex IV.

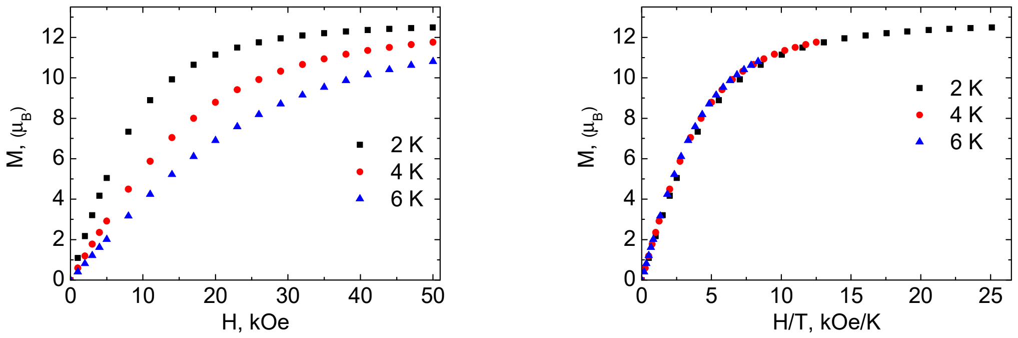

Figure S14. The M(H) and M(H/T) dependences at different temperatures for complex VII.

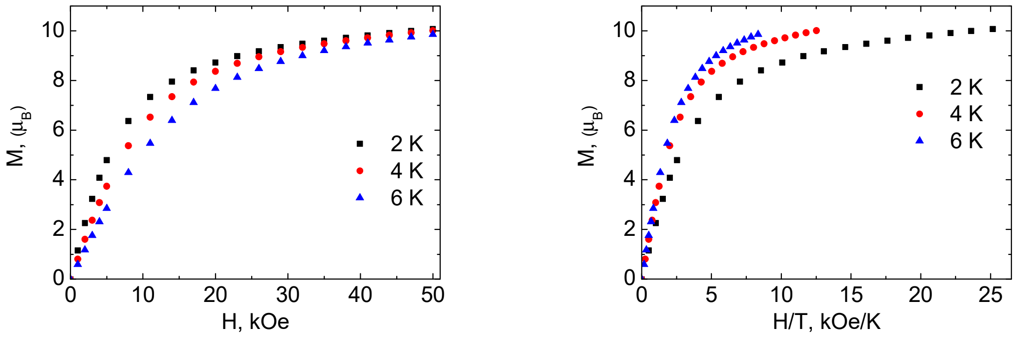

Figure S15. The M(H) and M(H/T) dependences at different temperatures for complex VIII.

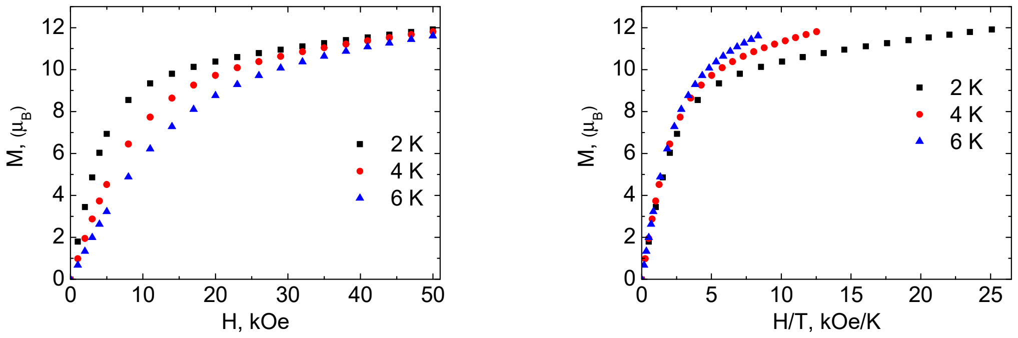

Figure S16. The M(H) and M(H/T) dependences at different temperatures for complex IX.

Table S7. Fit of χ_MT(T) dependence for II calculated by PHI developed by N.F. Chilton et al.

T, K	$\chi_M T$, cm^3 K mol^{-1}
299.636	16.362
284.387	16.3624
269.105	16.3629
253.744	16.3634
238.417	16.3641
223.079	16.3648
207.494	16.3656
192.155	16.3665
176.82	16.3676
161.472	16.3688
146.143	16.3703
130.788	16.3721
115.465	16.3744
100.199	16.3772
84.9443	16.3809
80.0239	16.3823
75.0369	16.3839
70.0394	16.3857
65.0408	16.3877
60.0399	16.3899
55.0318	16.3924
50.0298	16.3953
45.0281	16.3985
40.0277	16.4022
35.0418	16.4062
30.0288	16.4104
25.0139	16.4141
20.0184	16.4149
18.0111	16.4129
16.0108	16.408

14.0083	16.3978
11.9995	16.3773
10.0039	16.3362
8.99928	16.3006
7.99995	16.2478
6.99378	16.1659
5.98753	16.0344
4.98671	15.813
3.98355	15.4105
2.98078	14.6106
1.9748	12.8304

Table S8. Fit of χ_MT(T) dependence for VII calculated by PHI developed by N.F. Chilton et al.

T, K	$\chi_M T$, cm^3 K mol^{-1}
299.671	16.1501
284.305	16.1505
269.064	16.151
253.746	16.1515
238.394	16.152
223.068	16.1527
207.536	16.1534
192.236	16.1542
176.842	16.1552
161.542	16.1563
146.195	16.1577
130.813	16.1594
115.528	16.1614
100.244	16.1639
84.981	16.1673
80.0449	16.1686
75.0499	16.17
70.0503	16.1717
65.0492	16.1735
60.0426	16.1755
55.0408	16.1778
50.0334	16.1804
45.0318	16.1833
40.0316	16.1866
35.0338	16.1902
30.0268	16.194
25.0163	16.1974
20.0238	16.198
18.0152	16.1962
16.0105	16.1916
14.0058	16.1822
12.0026	16.1635
10.0026	16.1259
9.0007	16.0935
7.99927	16.0453
6.99743	15.9711
6.0011	15.8529
4.99389	15.6509
3.99272	15.2861
2.99222	14.5603
1.98526	12.9247

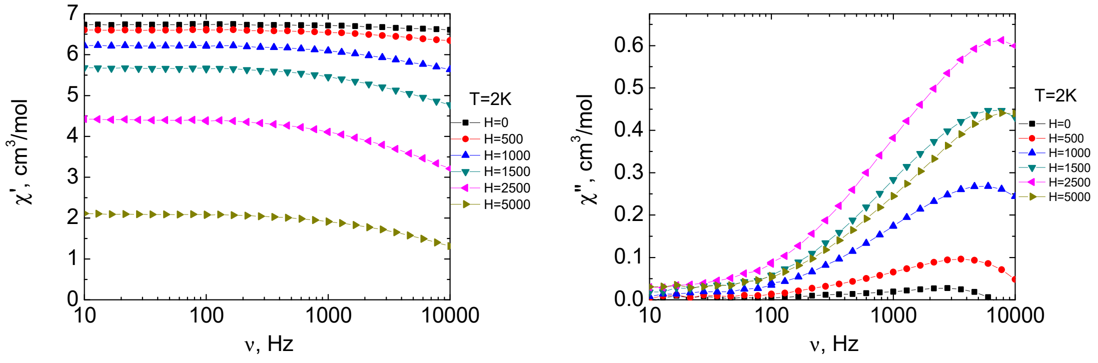

Figure S17. Frequency dependencies of real, χ' (left) and imaginary, χ'' (right) components of dynamic magnetic susceptibility for complex II at T = 2 K under various dc magnetic fields. Solid lines are visual guides.

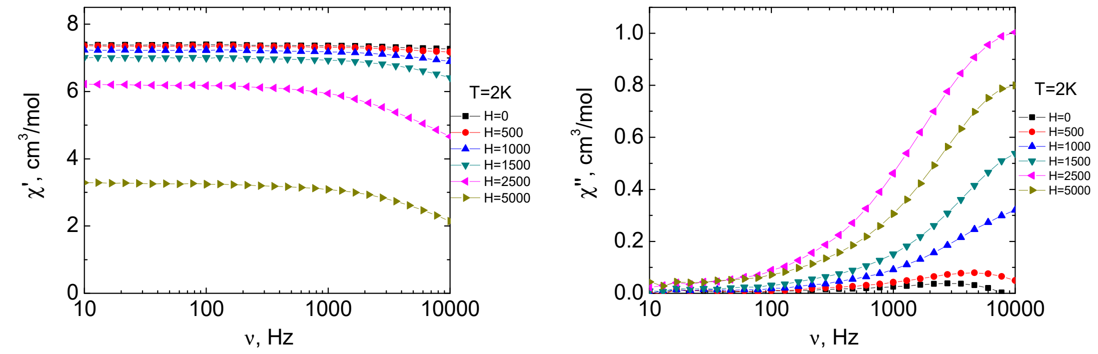

Figure S18. Frequency dependencies of real, χ' (left) and imaginary, χ'' (right) components of dynamic magnetic susceptibility for complex III at T = 2 K under various dc magnetic fields. Solid lines are visual guides.

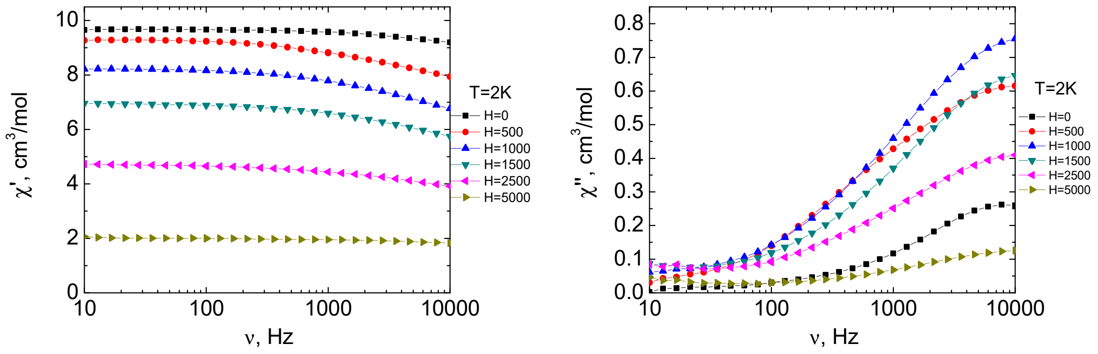

Figure S19. Frequency dependencies of real, χ' (left) and imaginary, χ'' (right) components of dynamic magnetic susceptibility for complex IV at T = 2 K under various dc magnetic fields. Solid lines are visual guides.

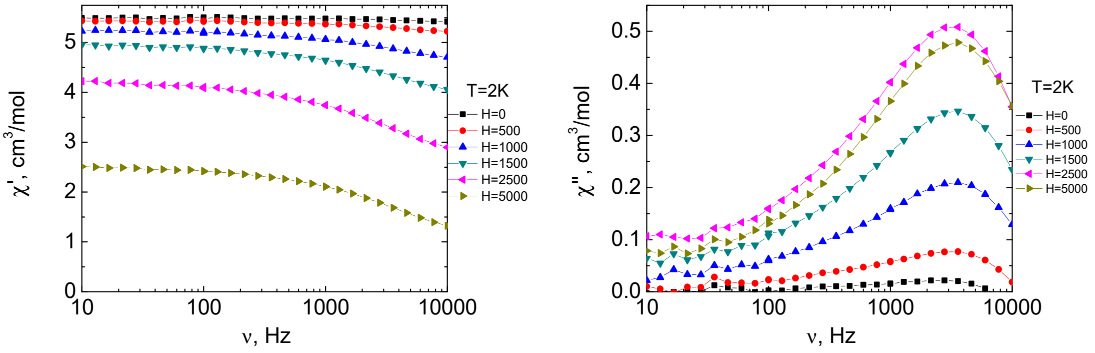

Figure S20. Frequency dependencies of real, χ' (left) and imaginary, χ'' (right) components of dynamic magnetic susceptibility for complex VII at T = 2 K under various dc magnetic fields. Solid lines are visual guides.

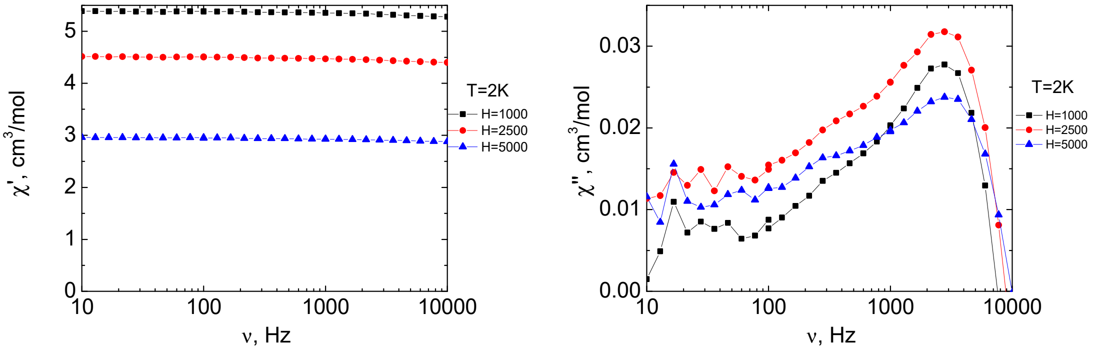

Figure S21. Frequency dependencies of real, χ' (left) and imaginary, χ'' (right) components of dynamic magnetic susceptibility for complex VIII at T = 2 K under various dc magnetic fields. Solid lines are visual guides.

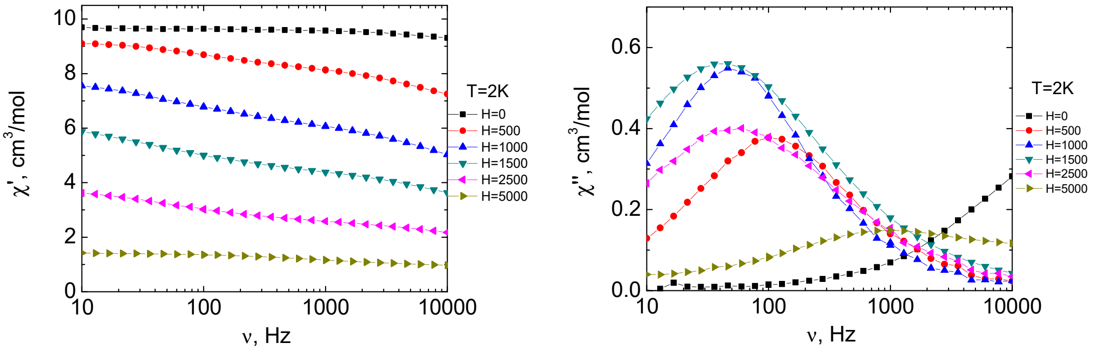

Figure S22. Frequency dependencies of real, χ' (left) and imaginary, χ'' (right) components of dynamic magnetic susceptibility for complex IX at T = 2 K under various dc magnetic fields. Solid lines are visual guides.

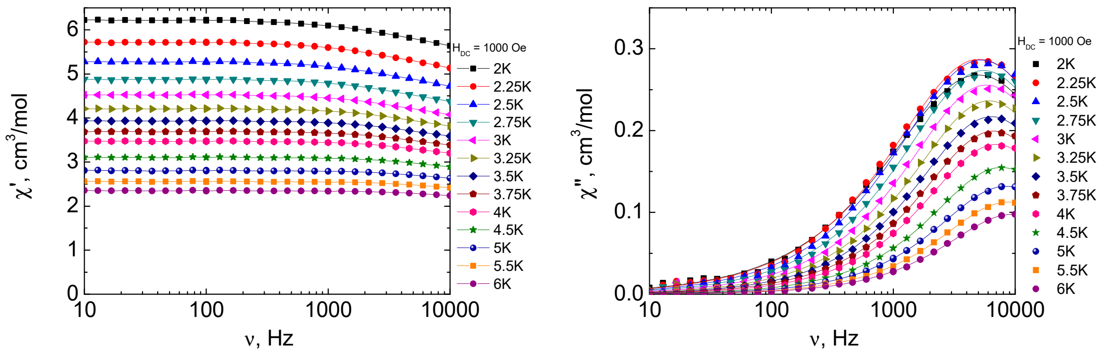

Figure S23. Frequency dependencies of the real (left) and imaginary (right) components of the ac-magnetic susceptibility for complex II in the 2–6 K range taken under the optimal 1000 Oe dc-field. Solid lines represent fitting by the generalized Debye model.

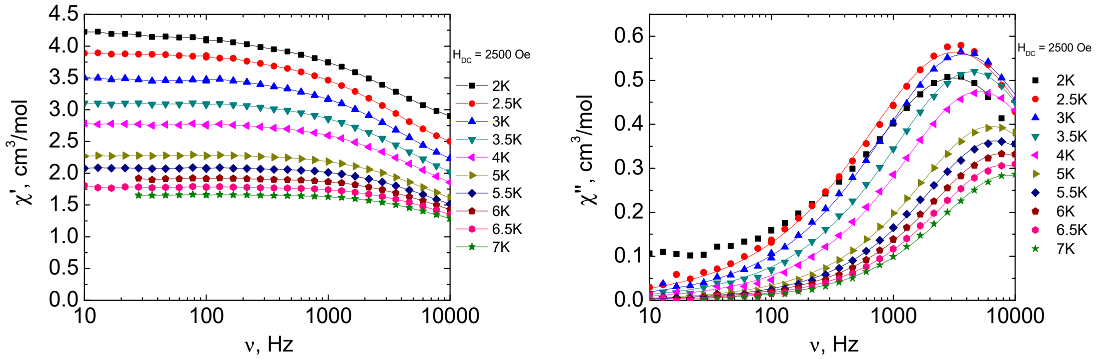

Figure S24. Frequency dependencies of the real (left) and imaginary (right) components of the ac-magnetic susceptibility for complex VII in the 2–7 K range taken under the optimal 2500 Oe dc-field. Solid lines represent fitting by the generalized Debye model.

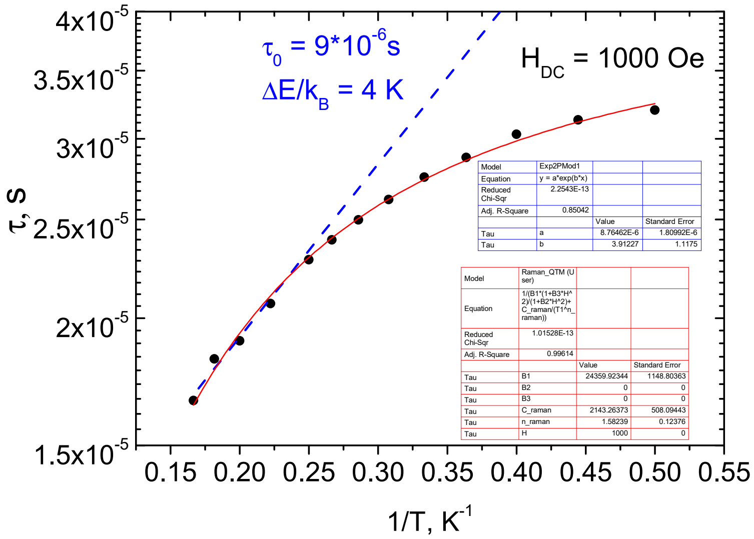

Figure S25. τ *vs.* 1/T plot for complex II under H_{dc} field of 1000 Oe at T = 2 K. Blue dashed line represents fitting by Orbach mechanism (Arrhenius equation). Solid red line represents fitting by QTM+Raman relaxation mechanisms.

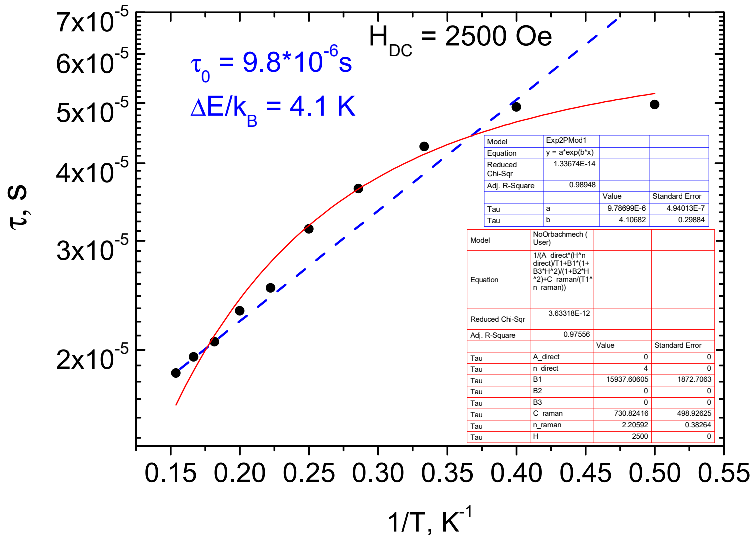

Figure S26. τ *vs.* 1/T plot for complex VII under H_{dc} field of 2500 Oe at T = 2 K. Blue dashed line represents fitting by Orbach mechanism (Arrhenius equation). Solid red line represents fitting by QTM+Raman relaxation mechanisms.

© 2020 by the authors. Submitted for possible open access publication under the terms and conditions of the Creative Commons Attribution (CC BY) license (http://creativecommons.org/licenses/by/4.0/).
